# Recent Advances on Nitrofluorene Derivatives: Versatile Electron Acceptors to Create Dyes Absorbing from the Visible to the Near and Far Infrared Region

**DOI:** 10.3390/ma11122425

**Published:** 2018-11-30

**Authors:** Guillaume Noirbent, Frédéric Dumur

**Affiliations:** Aix Marseille Univ, CNRS, ICR UMR 7273, F-13397 Marseille, France

**Keywords:** fluorene, nitrofluorene, Knoevenagel reaction, near infrared absorption, push–pull chromophore, poly(nitro)fluorene

## Abstract

Push–pull dyes absorbing in the visible range have been extensively studied so that a variety of structures have already been synthesized and reported in the literature. Conversely, dyes absorbing in the near and far infrared region are more scarce and this particularity relies on the following points: difficulty of purification, presence of side-reaction during synthesis, low availability of starting materials, and low reaction yields. Over the years, several strategies such as the elongation of the π-conjugated spacer or the improvement of the electron-donating and accepting ability of both donors and acceptors connected via a conjugated or an aliphatic spacer have been examined to red-shift the absorption spectra of well-established visible dyes. However, this strategy is not sufficient, and the shift often remains limited. A promising alternative consists in identifying a molecule further used as an electron-accepting group and already presenting an absorption band in the near infrared region and to capitalize on its absorption to design near and far infrared absorbing dyes. This is the case with poly(nitro)fluorenes that already exhibit such a contribution in the near infrared region. In this review, an overview of the different dyes elaborated with poly(nitro)fluorenes is presented. The different applications where these different dyes have been used are also detailed.

## 1. Introduction

**Figure 1:**
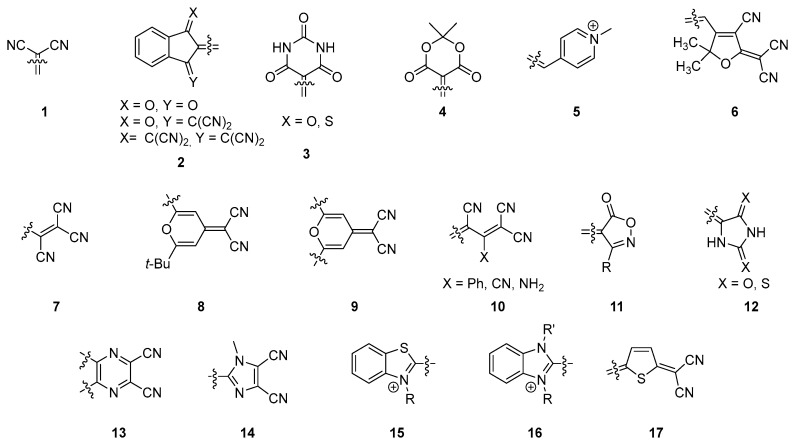
Chemical structures of common electron acceptors.

The reciprocal influence of an electron-donating and an electron-accepting group is capable to give rise to structures with fascinating properties. Optically, the first manifestation of this mutual interaction is a color change when the two partners are mixed, resulting in the presence of an additional absorption band which is not detectable in the absorption spectra of the initial partners considered separately. This absorption band corresponds to the charge transfer (CT) interaction where part of the electronic density is transferred from the electron-rich to the electron-deficient partner. Depending on the fact that the two moieties are (or not) connected to each other, this CT transition can be intermolecular or intramolecular. Historically, intermolecular charge transfer complexes have been extensively studied, focused on the metallic conductivity resulting from the grinding of two insulating organic compounds, i.e., tetrathiafulvalene (TTF) and tetracyano-quinodimethane (TCNQ) [1]. Following this pioneering work, the possibility to develop superconductors at low temperatures has driven extensive research efforts, and the emergence of the Bechgaard salts [2]. If these structures are attractive for their electrical properties, the resulting charge transfer complex renders these structures insoluble, drastically limiting the scope of applicability. Conversely, materials exhibiting an intramolecular charge transfer (ICT) i.e., meaning that the electron donor is connected to the electron acceptor by mean of a spacer (aliphatic or conjugated) are not necessarily insoluble so that these structures found widespread applications ranging from dye-sensitized solar cells (DSSCs) [3], nonlinear optical applications [4,5], solvatochromic probes [6,7,8], photochromes [9], single component semiconductors [10,11,12], electrochromes [13,14], and piezochromes [15]. Typically, these compounds are composed of a π-conjugated system with an electron-donating, and an electron-accepting part connected at both sides of the conjugated spacer [16,17,18,19]. If the number of groups used as electron donors cannot be calculated anymore in regards to the diversity of structures, the number of electron acceptors that can be covalently linked to electron donors is more limited, as exemplified by the list of molecules 1–17 presented in the Figure 1. Notably, malononitrile **1** [20], indanedione derivatives **2** [21], (thio)barbituric derivatives **3** [22], Meldrum derivatives **4** [23], pyridinium **5** [24], methyl-containing tricyanofurans **6** [25], substituted tricyanopropenes **7** [26], pyran derivatives **8** and **9** [27,28], 1,1,3-tricyano-2-substituted propenes **10** [29], isoxazolones **11** [30], hydantions and rhodanines **12** [31], pyrazines **13** [32], dicyanoimidazoles **14** [33], benzo[*d*]thiazoliums **15** [34], benzo[*d*]imidazoliums **16** [35], and dicyanovinyl-thiophen-5-ylidenes **17** [36] can be cited as the most common acceptors.

**Figure 2:**
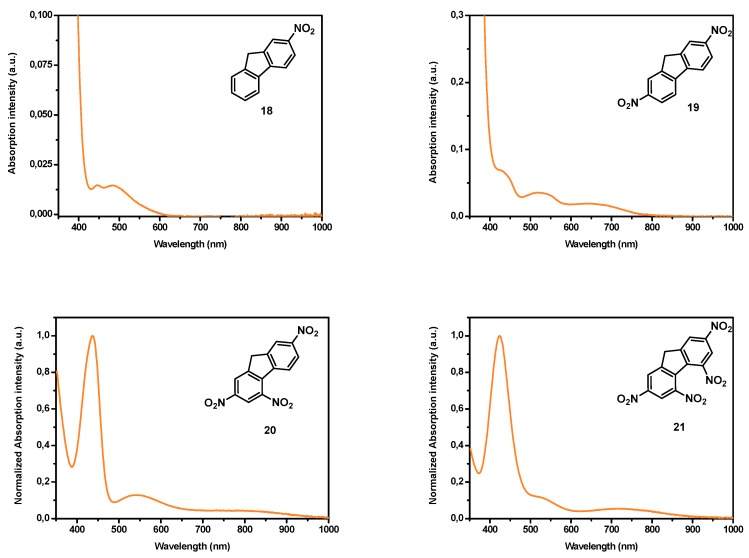
UV-visible absorption spectra of **18**–**21** in *N*,*N*-dimethylformamide (DMF).

**Figure 3:**
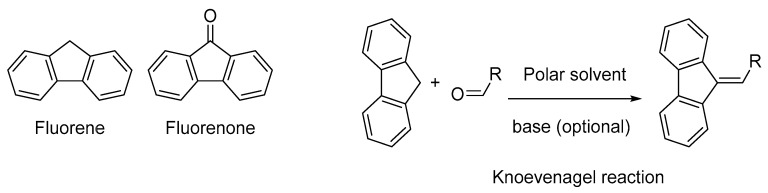
Chemical structures of fluorene and fluorenone.

For all these acceptors and irrespective of the electron donors, the intramolecular charge transfer band is centered in the visible range and this latter can be displaced towards the near infrared region if the length of the π-conjugated spacer introduced between the two partners is extended as much as possible. However, the extension of the π-conjugated spacer is often a hard work from a synthetic point of view and alternatives are actively researched [37]. Considering that for numerous applications, an absorption in the near-infrared region is required and face to the fact that all the above-mentioned electron acceptors are not sufficiently electro-deficient to inherently position the charge transfer band in the near infrared (NIR) region without taking recourse to the elongation of the π-conjugated spacer, electron acceptors already displaying an absorption band in the NIR region are actively researched. In this field, and to the best of our knowledge, only (poly)nitrofluorenes such as dinitrofluorene **19** [38], trinitrofluorene **20** and tetranitrofluorene (TNF) **21** exhibit such an absorption band (see Figure 2). It has to be noticed that the absorption band detected in the NIR for **19**–**21** is not detectable in **18**. Based on this unique property, numerous push–pull dyes displaying an absorption peak in the NIR region have been designed. If (polynitro)fluorenone derivatives have been extensively studied for the design of intermolecular charge transfer complexes [39], the presence of the carbonyl function in fluorenone totally impede this structure to be used for the design of push–pull molecules by connecting the electron withdrawing/releasing groups to the carbon inserted between the two aromatic rings. Fluorenone derivatives will not be discussed in this review (see Figure 3). Indeed, by the presence of the activated CH_2_ group standing between the two aromatic rings, poly(nitro)fluorene derivatives constitute candidates of choice for the synthesis in one step of push–pull molecules by a Knoevenagel reaction, when opposed to an electron donor comprising an aldehyde function (see Figure 3).

As an interesting feature, when substituted with electron-withdrawing groups such as nitro groups, the methylene group standing between the two aromatic rings is sufficiently acid so that the Knoevenagel reaction on **20** and **21** can be carried out without any base, in non-toxic and polar solvents such as *N*,*N*-dimethylformamide (DMF). Principles of Green Chemistry can thus be applied to the synthesis of push–pull molecules (see Figure 3). Conversely, the methylene group in **18** and **19** is often not sufficiently activated to allow the Knoevenagel reaction to be carried out without a base and piperidine or pyridine are classically used.

In this review, an overview of the different push–pull structures comprising (poly)nitrofluorenes as electron acceptors is presented. More precisely, four different aspects will be detailed. First, numerous push–push dyes have been obtained by functionalization of the 9-position of the fluorene acceptor. Second, a series of dyes has also been obtained by nucleophilic substitution of secondary amines on cyano-substituted fluorenes and this second approach constitutes the second most widely used synthetic procedure to access to poly(nitro)fluorene-based dyes. Third, the conceptual unimolecular rectifier proposed by Aviram and Ratner has been a source of inspiration for the design of numerous chromophores and a series of dyes has been designed based on this proposed structure. Finally, parallel to organic donors, organometallic donors have also been investigated, and it constitutes the last part of this review. To end, and parallel to the synthesis and the examination of the different photophysical properties of the dyes, the different applications justifying the design of these structures will be detailed.

## 2. Push–Pull Molecules Based on the Connection of Strong Electron Donors at the 9-Position of the Fluorene Acceptors

**Figure 4:**
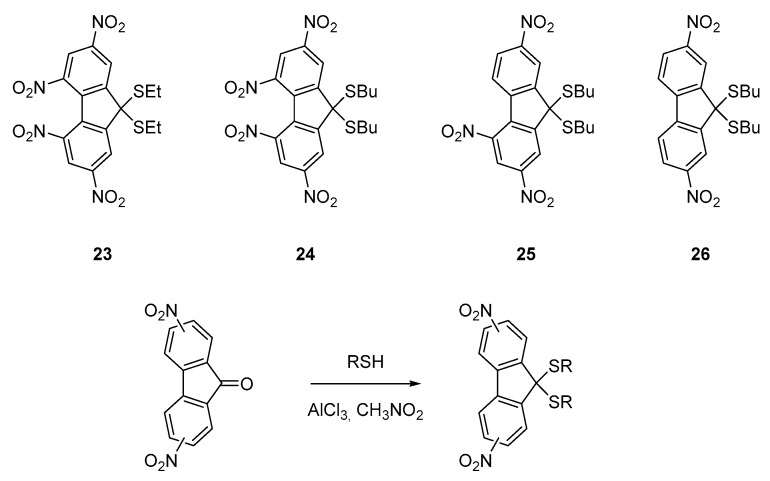
First push–pull molecules **23**–**26** based on poly(fluorene) acceptors.

The first report mentioning the design of push–pull molecules with poly(nitrofluorenes) **23**–**26** was published in 1984 [40]. In this pioneering work, relatively weak electron donors were used since alkylthio groups were employed. By virtue of the electron-donating ability of the sulfur atom, an absorption extending until 480 nm could be obtained for **25** and **26**. Despite the weak electron-donating ability of alkylthiols, the strong electron-accepting ability of **19**–**21** could be evidenced, the standard conditions of thioacetalization of ketones being unable to provide the targeted molecules. Indeed, the charge transfer interactions existing between the alkylthiols and nitrofluoren-9-one derivatives hampered the classical condensation procedures to provide the push–pull structures **23**–**26** (See Figure 4).

The four molecules could only be obtained using an unusual procedure making use of aluminium chloride as a mediator for the reaction [41]. Using this strategy, **23**–**26** could be obtained with reaction yields ranging from 85 to 93%. Interestingly, a weak charge transfer interaction could be evidenced for **23**–**26**, the lowest-energy transition exhibiting an onset at 470 nm for **25** and **26** and 410 nm for **23** and **24** respectively. Weakness of the interaction between the sulfur donor and the nitro acceptors and thus the low electronic delocalization in the ground state was confirmed by cyclic voltammetry, all molecules exhibiting redox potentials comparable to that obtained for alkyl 2,4,5,7-tetranitrofluorene-9,9-dipropionates that does not possess any electron-donating groups. These molecules possessing good electron acceptors connected to electron donors can efficiently sensitize the photoconductivity of semiconducting polymers by forming charge transfer complexes with these latter, especially with carbazole-containing polymers [42,43,44], and this ability was examined with poly(*N*-vinylcarbazole) (PVK) which is a standard semiconducting polymer. This interaction was demonstrated by using two different techniques i.e., the space-charge-limited xerographic discharge and by an electrochemical time-of-flight (ETOF) method. It has to be noticed that the first commercially available organic photoconductor was based on a charge-transfer complex between tetranitrofluorene (TNF) and PVK, justifying the different studies devoted to this topic. As interesting feature, the spectral zone of photoconductivity of the carbazole-containing polymers can be selected by the appropriate choice of the push–pull molecule. By comparing the electron mobilities of **23**–**26** with that of the reference 2,4,7-trinitrofluoren-9-one, comparable transport properties could be determined [45,46,47,48], demonstrating the pertinence of the strategy for the design of photoconductive materials with poly(nitro)fluorene derivatives.

**Figure 5:**
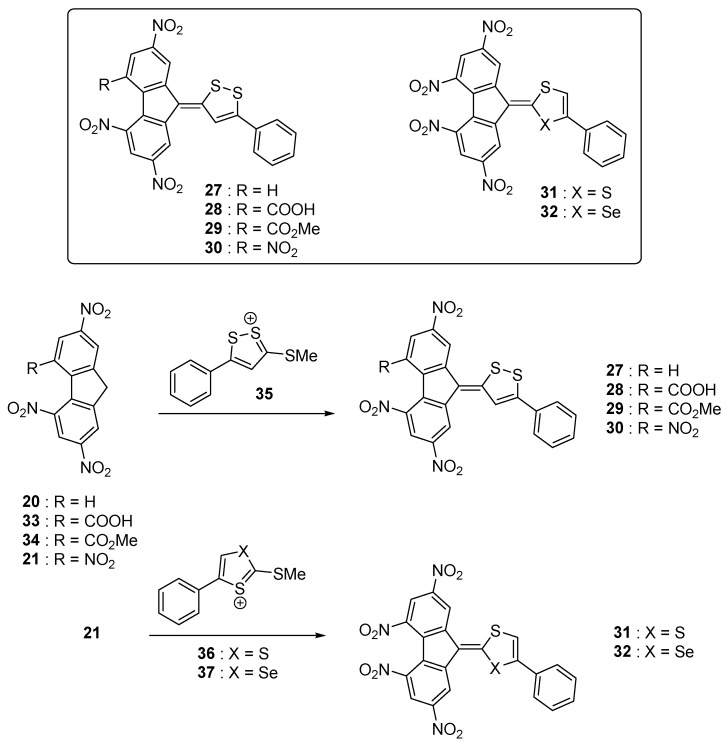
Push–pull dyes **27**–**32** comprising dithiolylidene or selenathiolylidene electron donors.

In contrast, by improving the electron donating ability of the donors, a significant red-shift of the absorption combined with the appearance of a second and new long-wavelength absorption band can be detected at lower energy than the intramolecular charge transfer. This specific band could be evidenced with the series of push–pull molecules **27**–**32** (see Figure 5) [49]. This additional absorption band is really a characteristic from the poly(nitro)fluorene acceptors. As electron donors, dithiolylidene and selenathiolylidene groups were used, these groups being among the best electron donors [50,51]. **27**–**32** were obtained by condensation of the corresponding dithiolylium **35**, **36** and selenathiolylium salts **37** with the electron acceptors **20**, **21**, **33** and **34** [52,53]. More precisely, due to the presence of multiple electron-accepting nitro groups on **20** and **21**, these latter are strong C-H acids [54] allowing the condensation in a highly polar solvent such as DMF to be carried out without any base. From a photophysical point of view, two charge transfer bands could be detected for the whole series **27**–**32**, the first one being detected between 446 and 500 nm and the second one between 587 and 633 nm respectively. Almost no influence of the heteroatom on the electronic transitions of **31** and **32** could be evidenced (see Table 1).

In sharp contrast, positions of the sulfur atoms clearly influence the ICT bands and a blue-shift of 60 nm for **31** relative to that of **30** was determined. Considering that the same electron acceptor is used for **30** and **31** (i.e., **21**), the enhanced electron donating ability of 1,2-dithiol-3-ylidene compared to that of 1,3-dithiol-2-ylidene was demonstrated. As attended, improvement of the electron accepting ability of the substituents in **27**–**30** resulted in a bathochromic shift of the ICT bands (see Table 1) and a bathochromic shift of the absorption maximum was also determined by increasing the solvent polarity. It thus evidences the higher polarity of the excited state for these compounds relative to that of their ground states. Intramolecular nature of the charge transfer was proved by protonation of the different molecules in sulfuric acid, resulting in the disappearance of the two ICT bands and the formation of colorless acidic solutions.

**Figure 6:**
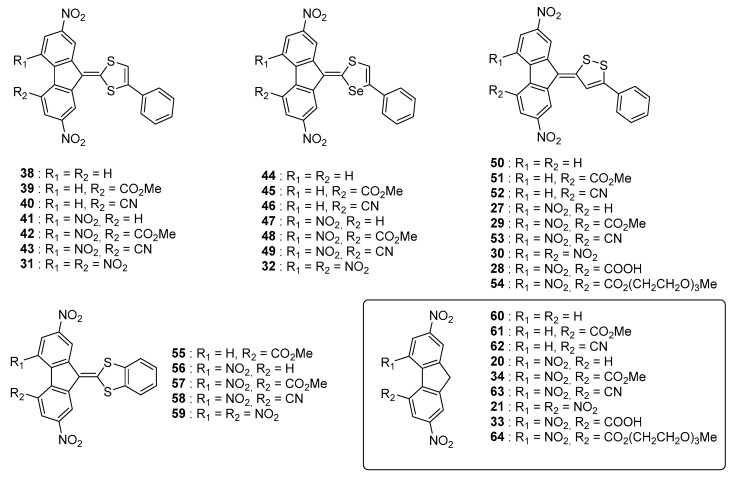
Push–pull dyes **27**–**32**, **38**–**59** using 1,2- and 1,3-dithiole, and 1,3-selenathiole as electron donors.

These different trends (lack of influence of the heteroatoms on the ICT maxima, positive solvatochromism) were confirmed in a subsequent study devoted to a series of 28 molecules (**27**–**32**, **38**–**59**) (See Figure 6) [55] Several molecules of the former study (namely **27**–**32**) were revisited in the context of this new work with aim at establishing a structure-photophysical properties relationship.

**Figure 7:**
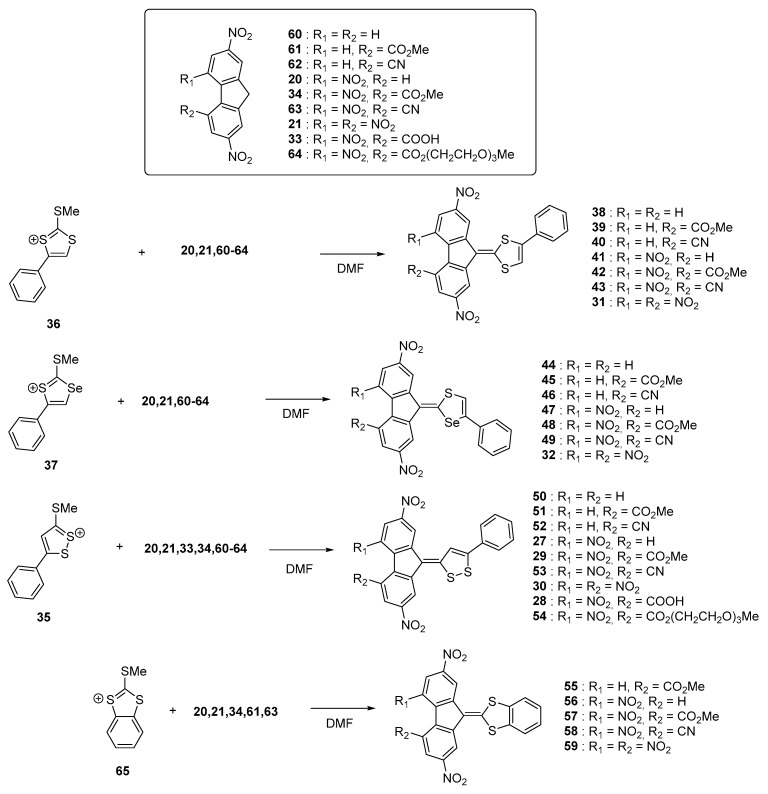
Synthetic routes to **27**–**32**, **38**–**59**.

Nine acceptors **20**, **21**, **33**, **34**, **60**–**64** were examined and the synthesis of the different dyes were carried out using procedures similar to that previously reported, using **35**–**37** and **65** as the electron donors. New conclusions could be determined from this work examining a wide range of structures. First, the reaction yields significantly decreased by reducing the electron-accepting ability of R_1_ and R_2_ so that use of a base was required while using **60**–**62** as acceptors (see Figure 7). As specificity, **60**–**62** are not substituted with nitro groups, deactivating the methylene group of the fluorene acceptor. The intramolecular nature of the charge transfer complex was proved by dissolving the different compounds in sulfuric acid, resulting in the formation of colorless solutions by protonation of the electron-donating part. Reversibility of the process was also evidenced upon addition of water, regenerating the initial compounds.

Only acidic sensitive compounds comprising nitrile or ester groups could not be entirely recovered, these groups being partially hydrolysed in strongly acidic conditions. Intramolecular nature of the charge transfer band was also proved by UV-visible spectroscopy, with a perfect and linear concentration dependence of the absorbance of their absorption maxima.

As other finding, modification of the linkage of the benzene ring to the donor group drastically alter of the absorption properties, with a hypsochromic shift of the absorption peaks of about 70 nm when the aromatic ring is fused to the donor (see series of compounds **55**–**59** vs. the series of molecules **27**–**30**, **50**–**54**). Examination of the solvatochromism proved to be quite complex, all the dyes exhibiting two intramolecular charge transfer bands originating from different transitions. (see Table 2).

Considering that the electronic transitions involved in these different absorption bands are not the same, the solvatochromic behaviors of the two bands varied separately and opposite behaviors were sometimes found for the two bands of a same dye. While using correlations based on the basicity and/or the polarity of the solvent such as the Koppel–Palm correlation, no linear relationships could be obtained [56,57]. A similar behavior was observed while using more traditional multi-parameter polarity scales such as the normalized Reichardt’s parameter (E^N^_T_) or the Dimroth–Reichardt E_T_(30) index [58]. Parallel to the solvatochromism, a thermochromism could be detected for all compounds, evidencing a modification of the solute-solvent interactions with the temperature [59,60]. Thus, a hypsochromic shift while increasing the temperature was determined for all compounds, with a more pronounced shift for the low energy ICT band. To illustrate this, a blue shift of 72 nm was observed in chlorobenzene for the second ICT band of **31** while increasing the temperature from 3 to 92°C whereas a blue shift of only 7 nm was detected for the high-energy transition.

To quantitatively examine the temperature effect, a linear dependence with inverse temperature could be evidenced. As attended, a negative halochromic behavior was detected, resulting from the protonation of the electron-donating dithiolylium. Interestingly, at certain concentration, an additional band in the 800–1000 nm region was detected for **31** and **32**, assigned to the formation of radical ion species. Precisely, the generation of radical species was assigned to bimolecular interactions between the neutral form regenerated upon dilution and the protonated form, resulting to an intermolecular electron transfer between the neutral and the cationic form, producing a radical and a radical cation following the Equation (1).

**31** + **31**-H^+^ ↔ **31**^+^ + **31**-H (1)

**Figure 8:**
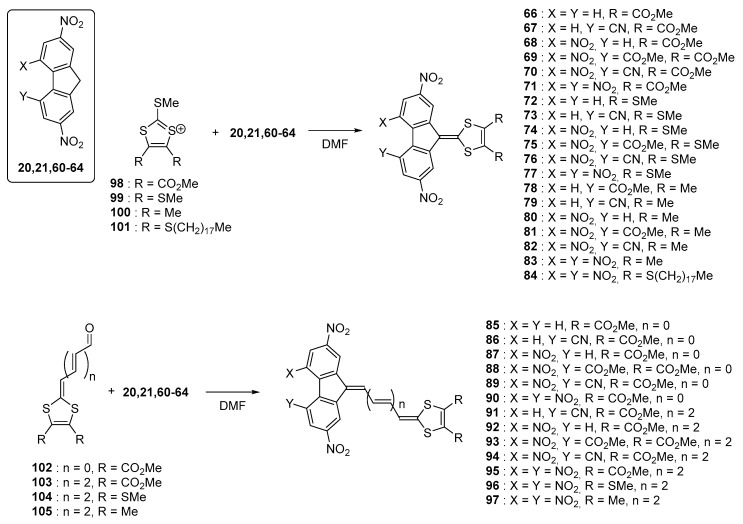
Synthetic routes to push–pull dyes **66**–**97** based on 1,3-dithiole donor moieties.

Electrochemical investigations revealed the different molecules to exhibit two redox processes, with two closely spaced reduction waves centered on the fluorene moieties, corresponding to the formation of the radical anion and dianion. For the series of compounds **32**, **44**–**49**, an additional reversible reduction process was detected at about −2.0 V (vs. Fc^0^/Fc^+^ couple) and this process was assigned to the formation of the radical trianion species. Investigation of the substitution effects on the fluorene acceptors revealed the difference between the two first reduction processes to be almost constant, whatever the substitution of the fluorene core was. Position of the ICT band is highly sensitive to the strength of the electron donors and acceptors that are selected for designing push–pull dyes but position of this transition can also be modified with lengthening the conjugated chain between the two partners. Influence of this parameter was notably examined with a series of chromophores bearing 1,3-dithiolylium donors [61]. The different dyes **66**–**97** were synthesized using a strategy comparable to that used for the former series (see Figure 8). **98**–**105** were used as the electron donors and **20**, **21**, **60**–**64** as the electron acceptors.

Upon elongation of the conjugated spacer, an extension of the highest occupied molecular orbital (HOMO) level over the donor part and the π-conjugated system is logically observed, resulting in a decrease of the HOMO-LUMO gap (where LUMO stands for lowest unoccupied molecular orbitals). Thus, comparison between **71**, **83**, and **90** revealed the second ICT band to shift from 544 to 588 and 636 nm respectively in 1,2-dichloroethane (see Table 3). Parallel to this, the molar extinction coefficient drastically increased from 9800 to 21000 and 39000 dm^3^·mol^−1^·cm^−1^ respectively upon elongation of the conjugated spacer. By electrochemistry, a cathodic shift could be detected (E_red_ (71) = −0.80 V, −1.02 V and E_red_ (83) = −0.83 V, −1.05 V vs. Ferrocene/Ferrocenium (Fc^0^/Fc^+^) couple), consistent with an improvement of the electron-donating ability in **83** and thus a lower ability for the fluorene moiety to accept electrons.

As potential application for these structures, the most soluble derivative **84** was examined as a sensitizer in photothermoplastic storage media (PTSM) based on poly[*N*-(2,3-epoxypropyl)carbazole] (PEPC) [44]. Notably, photothermoplastic materials can find numerous applications in aerospace and astrophysics [62]. In the present case, high values of holographic sensitivity in the ICT region were determined, outperforming the results obtained with tetranitrofluorene **21**, when used as the sensitizer for the same polymer. Especially **84** is a promising candidate for this application as it possesses a significant electron affinity (2.1 eV deduced from its reduction potentials (E_red1_ = −0.81 V, E_red2_ = −1.04 V vs. Fc^0^/Fc^+^ couple), facilitating the formation of charge transfer complexes with electron donors such as poly(carbazoles).

**Figure 9:**
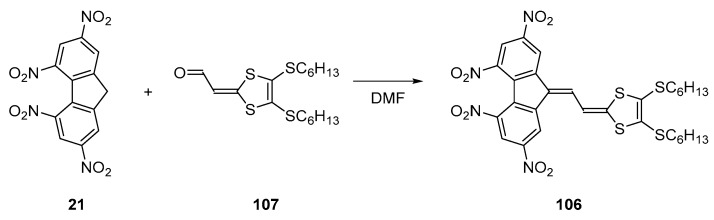
Synthetic routes to tetranitrofluorene (TNF)-based dye **106**.

Finally, the best comprise between electron affinity and intramolecular charge transfer energy was obtained with the molecule 106 [63]. This molecule was obtained by condensation of the aldehyde **107** with **21** in DMF (See Figure 9). Comparison of the electrochemical properties of this molecule with those of **21** evidenced the two molecules to exhibit similar redox potentials (−0.89 V, −1.04 V and −1.78 V for 106 vs. −0.88 V, −1.15 V and −1.79 V for 21 vs. Fc^0^/Fc^+^ couple respectively), providing similar electron affinities and thus electron accepting abilities to the two molecules. However, compared to its analogue **84**, a red-shift of the two ICT bands was obtained for **106**, with a red-shift of 95 and 33 nm for the two ICT bands and an increase of the peak intensity of 1.5 and 3 for the two ICT bands respectively (See Table 4). This enhancement of the molar extinction coefficient is consistent with the elongation of the π-conjugated system, increasing the oscillator strength and thus the electronic delocalisation upon excitation.

While comparing **84** and **106**, the ability of **106** to sensitize the photoconductivity of the carbazole-based polymer PEPK-1 was greatly enhanced, by the red-shift of its absorption bands but also by the similar intensity of the two ICT bands, widening the photoresponse over the 500–700 nm region. Indeed, the photoresponse of **84** was limited to the 550–650 nm region due to the presence of a unique ICT band.

**Figure 10:**
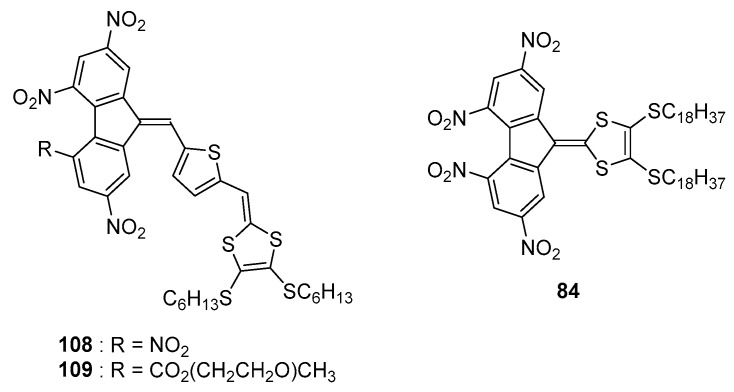
Chemical structures of **108**, **109** and **84**.

As already mentioned, elongation of the π-conjugated spacer is favorable to increase the molar extinction coefficient of the intramolecular charge transfer and to redshift the absorption maximum. An improvement of the photoconductivity sensitization is also attended if the electron affinity of the chromophore is enhanced, favouring the formation of a charge transfer complex with the sensitized polymer. With aim at optimizing these different parameters (length of the π-conjugated spacer, electron donating/accepting ability of the two partners), a thiophene was introduced between the electron-donating 1,3-dithiole group and the fluorene acceptor (see Figure 10) [64].

From a photophysical point of view, only few details were provided in this work, excepted that the two ICT bands are detected in the 400–900 nm region, with a significant red-shift of the absorption maximum accompanied by a substantial increase of the molar extinction coefficient for **108**/**109** relative to that of **84**. By electrochemistry, unexpectedly, an irreversible one-electron reduction process was observed for **108** and **109**, identified as resulting from the presence of the hydrogen on the double bond close to the acceptor moiety. As a consequence of this electrochemical irreversibility, when tested as sensitizers for the carbazole-based polymer PEPK, only a poor photoresponse in the ICT band of **108**/**109** was observed and this result was assigned to the poor photochemical stability of the radical anion previously demonstrated by the irreversibility of the reduction process. Solubility of the sensitizer is another major issue, especially for fluorene derivatives that are prone to aggregate, and this issue was addressed by elongating the chain (**109** vs. **108**). The higher solubility of **109** compared to that of **108** was evidenced.

**Figure 11:**
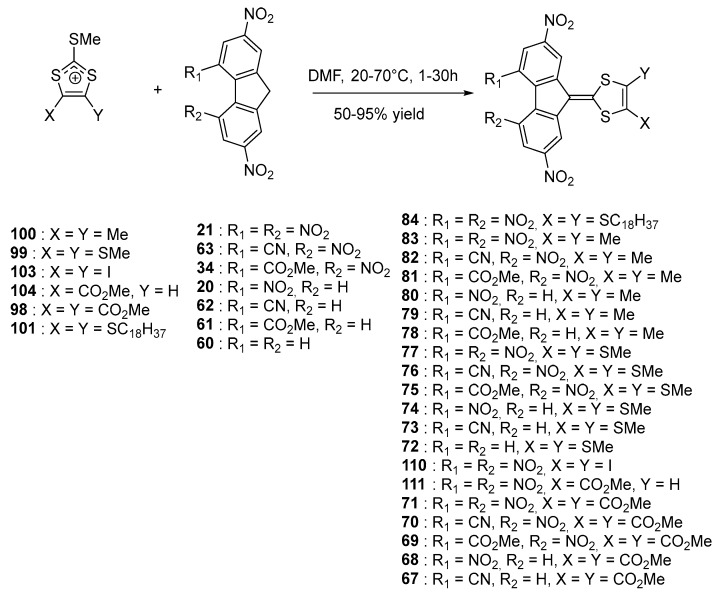
Synthetic route to **67**–**84** and **110**–**111**.

Recently, the design used for the synthesis of 84 was revisited in a more ambitious study where **21** structures were examined [65]. Their chemical structures are depicted in the Figure 11. In fact, compared to the previous study [61], only two new structures were added, namely **110** and **111**. However, a more detailed study was carried out in this new work.

All molecules **67**–**84** and **110**, **111** exhibited a strong color ranging from red to black and the different dyes were also characterized by a low solubility in most of the common organic solvents. This is the reason **84** was synthesized: in order to greatly improve the solubility of this chromophore. As the main characteristic of this series, the different molecules are characterized by multiple redox states, ranging from four to five redox states what is rarely observed for purely organic molecules [66,67,68]. Precisely, until four reduction processes could be detected for **71**, **70**, and **68**, what is also observed for fullerene derivatives [69]. Considering that upon reduction, the radical anion, the dianion, the radical trianion, and the tetraanion are centered on the fluorene moiety, a major influence of the substitution pattern was evidenced on these different reduction processes. A summary of the redox potentials is provided in the Table 5. Precisely, a linear correlation for the first and second reduction processes could be determined while using the Hammett constants which quantify the impact of the substitution pattern on the redox potentials [70]. No influence of the substitution pattern of the dithiole moiety on the reduction potentials was detected, evidencing the negative charge to be exclusively localized on the fluorene fragment. These results were confirmed by the theoretical calculations done on the different molecules, evidencing the LUMO level to be mostly centered on the fluorene moiety.

While examining the position of the ICT bands, classical trends were determined such as a clear bathochromic shift of the ICT band while improving the accepting ability of the fluorene moiety or by improving the electron donating ability of the 1,3-dithiole moiety. Here again, a linear correlation with the sum of the Hammett parameters was demonstrated, evidencing this series of molecules to exhibit a classical modification of the ICT band with the strength of the donors and the acceptors. The most redshifted absorption was detected for 83, with an ICT band peaking at 611 nm. Only a weak positive solvatochromism was determined for all molecules by increasing the solvent polarity, the variation of the ICT band being lower than 20 nm. This result is indicative of a highly polar ground state. Conversely, a pronounced thermochromic behavior was evidenced with a hypsochromic shift of the ICT band while increasing the temperature. This phenomenon was notably demonstrated in high-boiling point solvents such as chlorobenzene and 1,2-dichlorobenzene. Such a behavior is not so unusual since it was previously reported for polythiophenes [71,72]. Finally, among the series of 27 molecules, only **84** was examined as an electron acceptor for the preparation of CTC with carbazole-containing polymers. Precisely, 84 was used for recording holograms on photothermoplastic storage media (PTSM) materials in combination with poly(*N*-epoxypropylcarbazole) (PEPK). To reach the maximum sensitivity, a concentration of only 2 wt.% was required for **84** compared to 10 wt.% for benchmark sensitizers such as 2,4,7-trinitrofluorenone and 2,4,7-trinitro-9-dicyanomethylene-fluorene, as a result of a broader UV-visible absorption spectrum.

**Figure 12:**
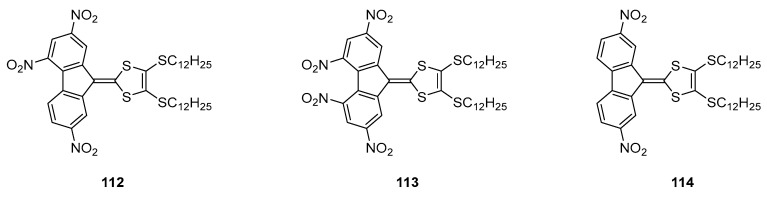
Chemical structures of **112**–**114**.

Until 2008, the unique application reported for poly(nitro)fluorene dyes concerned the electrophotographic processes with the sensitization of carbazole-containing polymers [73]. In 2009, the scope of applicability of poly(nitro)fluorene dyes was extended to aggregation-induced emission (AIE) and the design of red-emitting materials [74]. For this purpose, three molecules (**112**–**114**) were examined and the slight differences in chemical structures drastically impacted the optical properties and the supramolecular structures (See Figure 12). To examine this last point, symmetrically and asymmetrically substituted push–pull molecules were investigated.

While refluxing **112** and **113** in hexanes and cooling the solutions at 0 °C afforded in the two cases precipitates that were examined by optical microscopy. If the formation of spheres of 6–8 μm diameter and a nanostructured surface was observed for **112**, conversely, microtubes of 2.5–5 μm diameter were obtained for **113**. The size and morphology of the microstructures could be easily tuned by varying the cooling temperature. Thus, “flowers with petals” could be obtained while cooling the solution at 15 °C for **112**, replacing the microspheres previously obtained when the solution was cooled at 0 °C. By optically characterizing the microstructures, the two charge transfer bands detected in hexanes at 417 and 520 nm for **112** gradually evolved to a band at 480 nm with a shoulder at 520 nm upon aggregation. This new band was assigned to an intermolecular charge transfer between adjacent molecules in the solid state. Based on this finding, the cohesion between molecules in the solid-state results from intermolecular interactions between molecules, outperforming the intramolecular interactions. A similar behavior was evidenced for **113**, the intramolecular charge transfer band at 552 nm splitting into two new bands at 385 and 460 nm. While examining the photoluminescence properties, a 50-fold enhancement of the photoluminescence quantum yield (4.5%) was determined for **112** upon aggregation. An AIE process was thus evidenced. Conversely, the symmetrical substituted **113** did not show any enhancement of the photoluminescence properties upon aggregation. In fact, authors determined from the crystallographic investigations that the structure of 113 was more twisted than that of **112** in the solid state, quenching the fluorescence. Parallel to this, intermolecular interactions are responsible of the aggregation of **112** in the solid state whereas dipole interactions govern the packing of **113** in the solid state. It was thus concluded the molecular dipole moments in chromophores to be a crucial parameter governing the packing mode and the ability to design AIE emitters.

**Figure 13:**
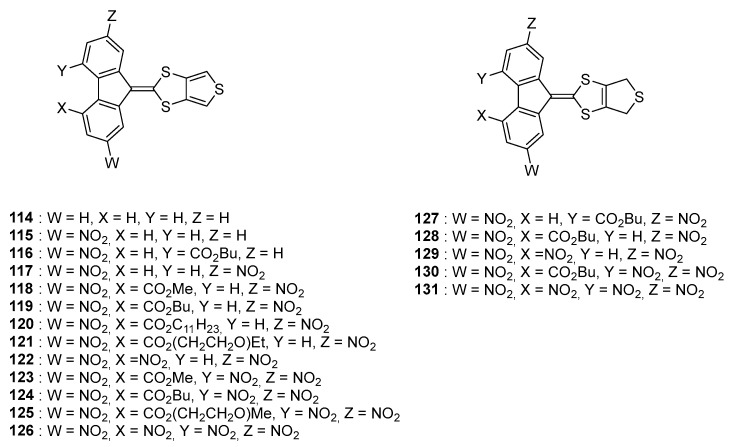
Structures of the different push–pull chromophores comprising a fused thiophene-1,3-dithiole **114**–**126** and dtetrahydrothiophene moieties **127**–**131**.

Parallel to 1,3-dithiole electron donors, thiophenes are also extensively used for the design of semiconducting polymers [75] and materials for NLO applications due to their exceptional electron donating ability and their oxidation potentials [76]. In this context, a series of poly(nitrofluorenes) where the thiophene group was not used as a spacer but as an electron donor were prepared [77]. More precisely, the 1,3-dithiole moiety was fused with the thiophene ring, furnishing an extended donor (see Figure 13).

For this series of dyes **114**–**131**, the synthesis of the acceptors and especially the selectivity during the mono- and dinitration reactions of the fluorene esters proved to be challenging and the reaction conditions had to be carefully optimized for each of them. To illustrate this, **138**–**142** could only be obtained by using a HNO_3_: AcOH 3:20 ratio starting from **133**–**137** whereas **143** and **144** could be more easily prepared while using a HNO_3_: AcOH 1:1 ratio. Conversely, **148**–**150** could only be obtained while reacting **133**–**135** in fuming nitric acid. In the case on long-chain esters (undecanyl and triethyleneglycol monomethyl ether and triethyleneglycol monoethyl ether), the purification of **145** and **146** was almost impossible due to the presence of a mixture of di- and trinitro derivatives that could not be separated due to similar polarities on the column chromatography. As an alternative strategy to access to **145** and **146**, the ester **144** was hydrolyzed in acidic conditions providing **153** and esterified with the appropriate alcohol furnishing the two expected esters **145**, **146** and even **154**.

**Figure 14:**
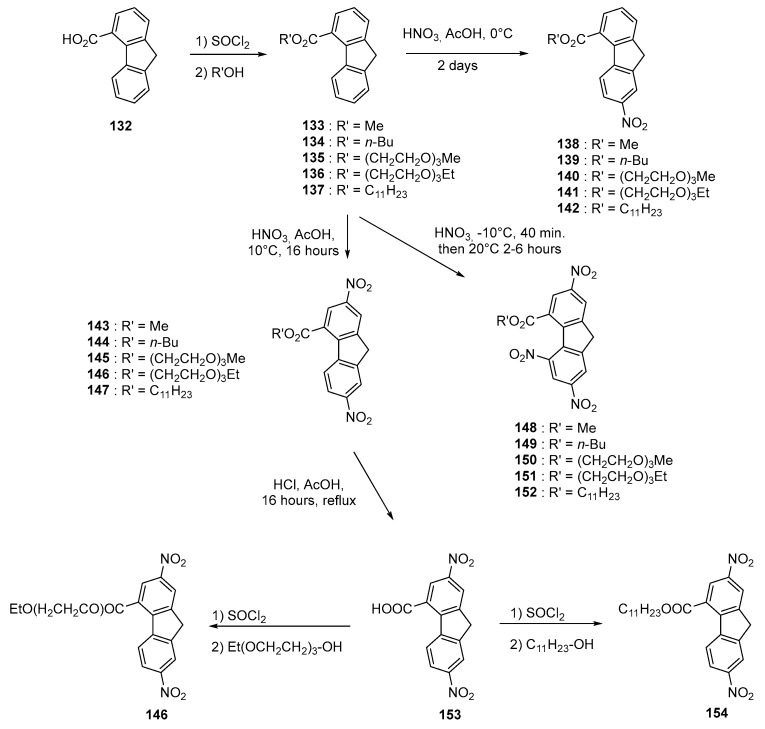
Synthetic routes to the various electron acceptors **132**–**154**.

As other surprising result, the synthesis of **151** could not be realized in the conditions used for **150**, the Nuclear Magnetic Resonance (NMR) analysis evidencing that the terminal ethyl group was lost (see Figure 14). Loss of the ethyl group and formation of a nitrate was confirmed by Infrared spectroscopy analyses. This unexpected behavior was not observed for **150**.

**Figure 15:**
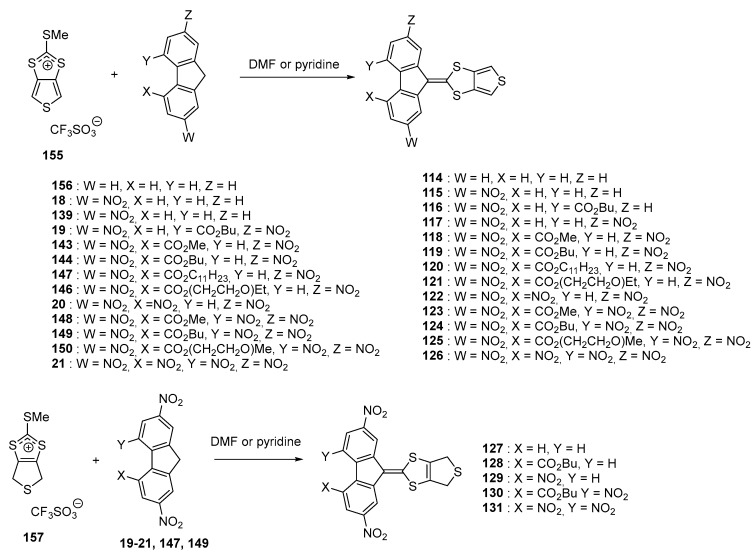
Synthetic route to the series of push–pull molecules **114**–**131**.

From a synthetic point of view, **114**–**126** were obtained in conditions adapted for each acceptor. Thus, due to the high reactivity of the tri- and tetranitrofluorene acceptors, condensation with salt **155** could be realized simply in DMF whereas pyridine was used as the solvent for the less reactive dinitro acceptors. Long reaction times were required for the reaction with mononitro derivatives, yielding the targeted molecules in low yields (40 to 76% yield) due to the degradation of the salt **155** over time. Compounds **114**–**116** could not be isolated pure. Face to this result, reactivity of the dihydrothiophene salt **157** towards condensation with fluorene acceptors was examined. Due to a greater delocalisation of the positive charge over the whole salt, reaction temperature for the synthesis of **127**–**131** could be considerably reduced (room temperature for the synthesis of **127**–**131** versus 100 °C for **114**–**125**, 50 °C for **126**) (see Figure 15). Reaction yields ranging from 47 to 64% were determined for the synthesis of **127**–**131**.

While examining the optical properties of the series of dyes **114**–**131**, a bathochromic shift of the two ICT bands was unexpectedly observed for **127**–**131** compared to that of their analogues **114**–**126**. This counter-intuitive behavior can be assigned to the fact that when the thiophene ring is fused to 1,3-dithiole, this latter doesn’t behave as a donor but as an acceptor, reducing the electron donating ability of the 1,3-dithiole moiety. A similar behavior was previously reported for TTF derivatives [78,79]. The most red-shifted ICT was detected for **131**, bearing **21** as the acceptor and the ICT was detected at 596 nm (see Table 6).

**Figure 16:**
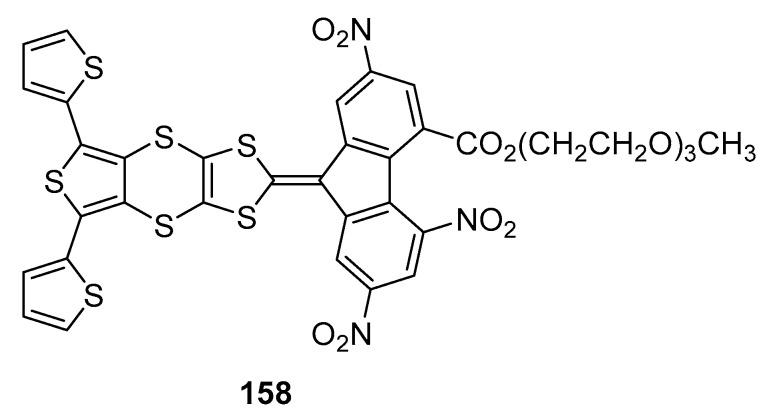
Chemical structure of **158**.

The electrochemical behavior of **114**–**131** revealed an irreversible oxidation process to take place upon oxidation. As deduced by UV-visible spectroscopy, the negative impact of the electron-withdrawing ability of the fused thiophene on the redox properties was confirmed, the oxidation potentials being detected at more cathodic potentials for the **114**–**126** vs. **127**–**131**. An oxidation process centered on the 1,3-dithiole fragment could be confirmed. Two closely-spaced reduction processes could be detected for all molecules with the presence of an additional reduction peak detected around −1.8 V (vs. Fc^0^/Fc^+^ couple), that was reversible, quasi-reversible or irreversible depending of the electron-withdrawing acceptor. No regular dependence with the substitution pattern of the fluorene moiety was evidenced for this third and additional peak. Finally, attempts to electropolymerize the series **114**–**126** failed, as a result of the formation of the radical cation onto the 1,3-dithiole part which is thus inappropriate for dimerization. The steric hindrance of the monomer was also suggested as impeding the monomeric units to connect to each other. This drawback was overcome with the terthiophene derivative **158** where two electroactive thiophene groups were introduced on both side of the thieno-1,3-dithiol-2-ylidene moiety (See Figure 16) [80]. In these conditions, a sufficient distance was introduced between the bulky fluorene units so that a polythiophene polymer could be formed by cycling between 0 and 1.40 V.

**Figure 17:**
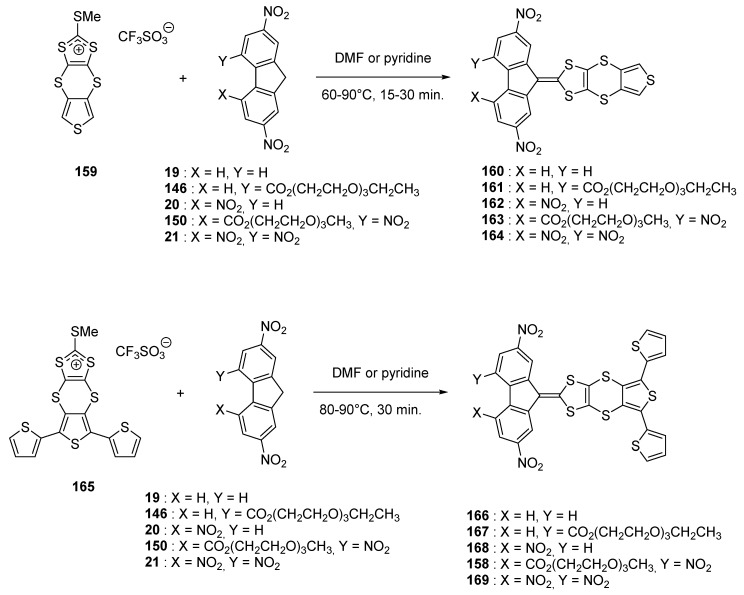
Structures of electropolymerizable monomers **160**–**164** and **158**, **166**–**169**.

To reduce the negative impact of the 1,3-dithiole moiety on the electrochemical oxidation of the thiophene moiety and the localization of the radical cation onto the 1,3-dithiole moiety, a strategy consisting in isolating electronically the two groups from each other was examined and in this aim, the **160**–**164** series was developed [81]. By analogy with the previous work, a series of molecules **158**, **166**–**169** where additional and lateral thiophene groups have been introduced to reduce the steric hindrance was also prepared (See Figure 17).

The different molecules were prepared using the standard procedure, by reaction consisting in the condensation of the substituted fluorenes with the appropriate dithiolium salt **159** or **165**. It has to be noticed that **165** is unstable so that it has to be used immediately after synthesis, in the next step, without purification. If **166** and **168** could be synthesized, their low solubilities impeded their purifications so that, even if obtained, these two compounds were thus further investigated.

By UV-visible spectroscopy, the low influence of the thiophene moiety as well as the 1,4-dithiino spacer on the charge transfer interaction was evidenced, especially by comparing the positions of the ICT bands with those of unsubstituted analogues previously reported (See Table 7) [55,61,82] Similarly, comparison of the oxidation potentials of the **167**–**169** series with those of the series **160**–**164** previously studied [77] evidenced the 1,4-dithiino spacer to decrease the electronic effects of the fluorene moiety on the oxidation ability of the thiophene unit, what is favorable for the oxidative electropolymerization of thiophene. Finally, for electropolymerization, only **158** was examined, this latter exhibiting the best compromise between solubility and electron-accepting ability of the fluorene fragment.

Poly(158) was obtained by electrochemical oxidation, by successive cycling between 0 and 1.4 V in dichloromethane. A dark blue polymer formed at the surface of the Gold electrode. By UV-visible spectroscopy, an ICT band located at 567 nm could be determined for the electrogenerated polymer. For comparison, poly(158) was also synthesized chemically, by oxidation with FeCl_3_, and an ICT band at the same position could be detected. By FTIR spectroscopy, analysis of the photoinduced IR spectrum of poly(158) revealed the presence of two representative infrared active vibration (IRAV) bands, one evidencing the presence of radical cations delocalized over the polythiophene backbone and a second one demonstrating the accepting part of the polymer to be localized over the fluorene units.

## 3. Push–Pull Molecules Obtained by Nucleophilic Substitution

**Figure 18:**
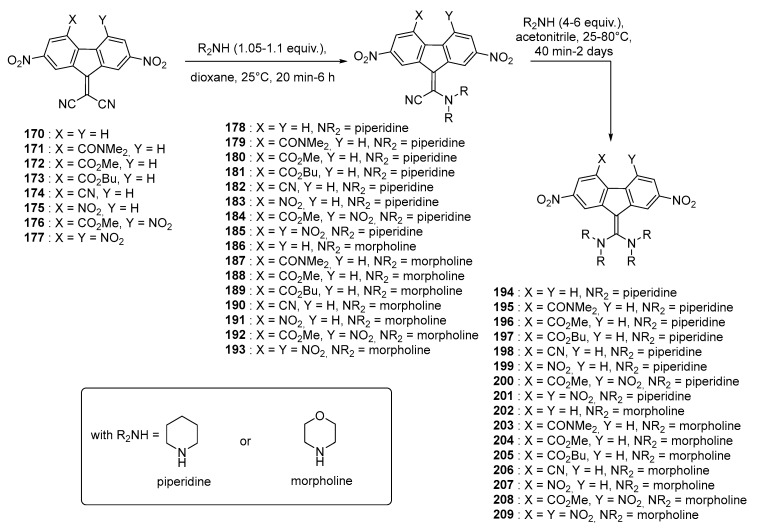
Structures of push–pull molecules **178**–**193** and **194**–**209**.

All the aforementioned chromophores have been obtained by functionalization of the methylene group of the fluorene acceptor. In this field, the most popular reaction has undoubtedly been the Knoevenagel reaction. Parallel to the Knoevenagel reaction, an alternative has also been proposed, based on the nucleophilic substitution on the dicyanomethylene fragment of dicyano-methylenefluorene acceptors by aliphatic amines. This reaction has previously been reported for strong electron acceptors such as tetracyanoquinodimethane (TCNQ) and tetracyanoethylene (TCNE) [83,84,85]. In the present case, one or two cyano groups can be substituted, depending of the reaction conditions. In 1995, Perepichka, et al. reported a series of push–pull molecules **178**–**193** and **194**–**209** prepared from **170**–**177**, based on the successive substitution of a cyano group by a secondary amine (See Figure 18) [86]. In this work, two different amines were investigated, namely piperidine and morpholine.

By the simultaneous presence of the electron donating aliphatic amino group and the electron-accepting fluorene fragment within the same molecule, appearance of an intramolecular charge transfer band located in the visible range could be detected. Interestingly, by NMR, a Z/E isomerization could be evidenced and by increasing the number of electron-withdrawing group on the fluorene fragment contributed to lower the rotation barrier. In this work, only few details were provided concerning the UV-visible absorption spectra of **178**–**193** and **194**–**209**. The unique informations concern the absorption range, going from 440 to 550 nm for **178**–**193** (440–550 nm) and 530–620 nm for **194**–**209** respectively. More efforts were devoted to determine the relationship existing between the position of the ICT band and the substituent effects. Especially, the Koppel–Palm four-parameters’ equation taking account from the polarity, the polarizability, the acidity and the basicity of the solvent was examined [56]. In this study, only the polarity and the basicity proved to be relevant to justify the shift with the substitution pattern.

**Figure 19:**
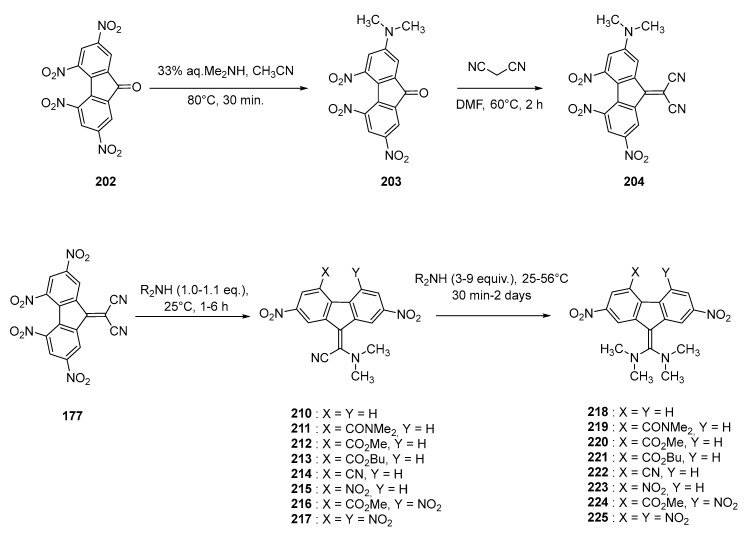
Structure of push–pull molecule **204** and the two series **210**–**217**, **218**–**225**.

Following this work, a more detailed study was proposed in 1996 by the same authors and the lateral functionalization of the fluorene core was investigated (see Figure 19) [82]. By electrochemistry, the specific behavior of **204** compared to the series **210**–**217** and **218**–**225** was evidenced. Notably, a difference of 560 mV was found between the two first reduction waves of **204** whereas this difference is only of 160 mV for **210**–**217** and **218**–**225** (see Table 8). The electron affinity (2.46 eV) of **204** was determined as being greater than that of the series **210**–**217** (1.65–2.03 eV) and the series **218**–**225** (1.36–1.85 eV). This result is counter-intuitive, considering the presence of the electron-donating group directly connected to the fluorene core but can rationalized on the basis of the sum of the nucleophilic constants. Considering that the two series **210**–**217** and **218**–**225** exhibit a strong absorption band in the visible range (see Table 8), the generation of the radical anion or the dianion of these structures should drastically reduce the electron accepting ability of the fluorene moiety, resulting in the disappearance of the intramolecular charge transfer band. A similar behavior should also be observed upon generation of the radical cation, making these structures appealing candidates for electrochromic applications. These conclusions were confirmed by spectro-electrochemical experiments, demonstrating a reduction of the ICT band upon oxidation and/or reduction of the chromophore, associated with the appearance of new bands corresponding to the formation of the radical cation, radical anion or dianion.

Finally, due to the broad absorption of these structures, the sensitization of the photoconductivity of carbazole-containing polymers was examined. While comparing the sensitization ability of **177**, **217** and **225**, a decrease in the order **177** > **217** > **225** was demonstrated, resulting from a reduction of the electron accepting ability of the fluorene core. An hypsochromic shift of the electrophotographic sensitivity was also observed, following the trend of the ICT band.

## 4. Push–Pull Molecules Based on the Aviram and Ratner Concept

**Figure 20:**
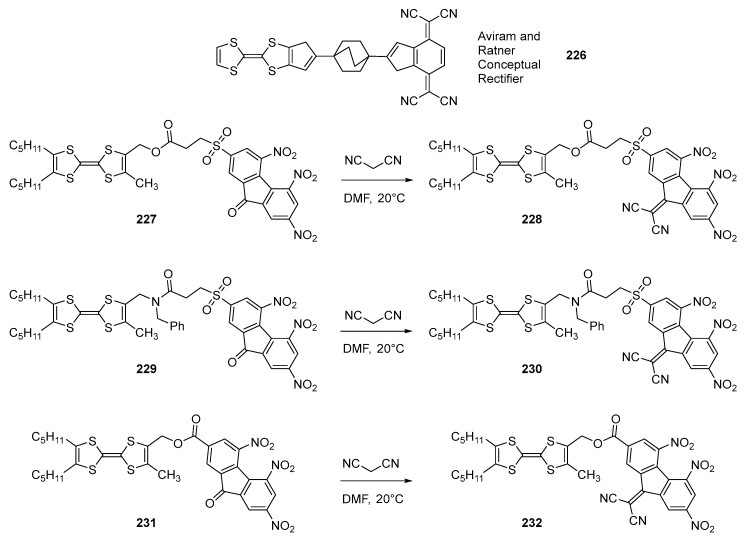
Structures of push–pull chromophores **227**–**232** derived from the Aviram and Ratner conceptual unimolecular rectifier **226**.

The unimolecular rectifier proposed by Aviram and Ratner in 1974 has excited during numerous years the imagination of researchers [87]. If proposed theoretically, numerous attempts have been carried out to synthesize this conceptual model **226**, without success [88,89,90]. The difficulty of synthesis lies in the combination within a unique molecule of a strong donor and a strong acceptor that will form an intermolecular charge transfer complex prior to the formation of the covalent bond [91,92,93,94]. If the initial concept was based on the covalent linkage of the remarkable electron donor tetrathiafulvalene (TTF) to the strong electron acceptor tetracyanoquinodimethane (TCNQ), numerous acceptors of lower electron-accepting ability were examined, as exemplified by quinones [95,96], tetracyanoanthraquinodimethane [89], fullerenes [48,97]. The conversion of weak acceptors to stronger acceptors subsequently to their covalent linkages has been extensively studied, but this conversion proved to be more difficult than anticipated and most of the attempts failed [88,89,90]. In 2002, Perepichka, et al. examined the combination of TTF with poly(nitro)fluorene acceptors. Three molecules were synthesized (See Figure 20) [98]. Here again, the strategy consisted in first connecting the fluorenone-based acceptors to the donors, providing the diads **227**, **229**, and **231**. In a second step, **227**, **229**, and **231** were converted to **228**, **230**, and **232** by reaction with malononitrile in DMF at room temperature. Concerning their designs, for **228** and **230**, the length of the flexible spacer was selected to be sufficient to allow an intramolecular electron transfer whereas a short spacer was selected for **232**, precluding the formation of an intramolecular interaction. Due to the use of strong electron donors and acceptors, an intramolecular electron transfer could effectively occur in **228** and **230** thanks to the use of a flexible spacer. The first manifestation of this ICT was the detection by EPR of a strong signal indicative of the formation of a radical determined as being the radical cation of TTF. Conversely, the radical anion of the acceptor was not detected, and this absence of signal was assigned to the formation of head to head dimers, quenching the signal. This behavior has previously been reported for TCNQ derivatives [99]. From an electrochemical point of view, diads **228**, **230** and **232** are characterized by a multiredox behavior, with the formation of the radical cation and dication of TTF and three reversible reduction processes centered on the fluorene moiety corresponding to the formation of the radical anion, dianion and radical trianion (See Table 9). Comparison of the redox potentials of **228**, **230** and the short-chain **232** revealed in this last case, the redox potentials of TTF not to be affected by the presence of the electron acceptor, and this was especially revealed by comparing the oxidation potentials of TTF in **231** and **232** (less than 10 mV of difference). Indeed, in this last case, **232** has been designed to preclude any intramolecular charge transfer and the two partners, in turn, behave as independent molecules. As final interesting feature, an extremely small HOMO-LUMO gap was determined for the three molecules, around 0.3 eV.

By UV-visible spectroscopy, the six push–pull molecules are characterized by two ICT bands, one in the visible range and another one in the NIR region. As anticipated, improvement of the electron-accepting ability resulted for **228** and **230** to a red-shift of the two absorption bands relative to that of **227** and **229**. Examination of the concentration dependence of the ICT band intensity was determined as being linear for **227**, **229**, **231** whereas a complete disappearance of the charge transfer band was detected for **231** and **232** for concentrations below 10^−4^ M. It can therefore be concluded that the two bands detected for **231** and **232** correspond to through-space interactions between adjacent molecules. Finally, **199** and **201** were examined for their electrochromic properties. **230** proved to be more promising than **228** as a partial decomposition was observed during cycling. This instability was partially assigned to the linker introduced between the donor and the acceptor, the ester connection being less stable than the amide connection. **230** proved to be an interesting material for photochromic applications as the two first oxidation and reduction processes are reversible, ensuring the regeneration of the initial molecule upon cycling.

## 5. Intramolecular Charge Transfer Complexes in Metal-Based Dyes

**Figure 21:**
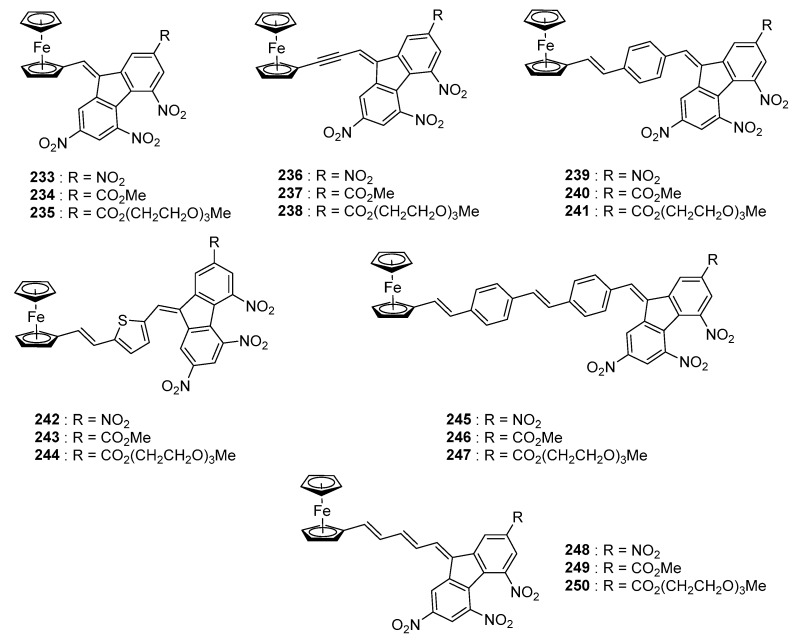
Push–pull chromophores **233**–**250** based on ferrocene.

Organic electron donors were not the only ones to be used for the preparation of push–pull chromophores comprising poly(nitro)fluorene acceptors. Ferrocene was also examined as a potential donor and influence of the length as well as the nature of the spacer introduced between the two fragments was studied (See Figure 21) [100].

For the series of metal complexes **233**–**250**, an absorption band ranging from 450 to 900 nm could be detected. These different complexes being designed for Non-Linear Optical (NLO) applications and in order to facilitate the determination of the quadratic hyperpolarizability by the electric field induced second harmonic (EFISH) generation, a soluble version of each complex was synthesized, namely, **235**, **238**, **241**, **244**, **247** and **250**. Going from **235** to **250**, the μβ values increased from 100 × 10^−48^ esu for **235** to 200 × 10^−48^ esu for **238**, 800 × 10^−48^ esu for **241** and 2400 × 10^−48^ esu for **250**. Evolution of the hyperpolarizability is consistent with the elongation of the π-conjugated spacer in these structures. While examining the optical properties, two ICT could be detected for all compounds. Only a minor variation of the maximum absorption was observed with the spacer (See Table 10).

**Figure 22:**
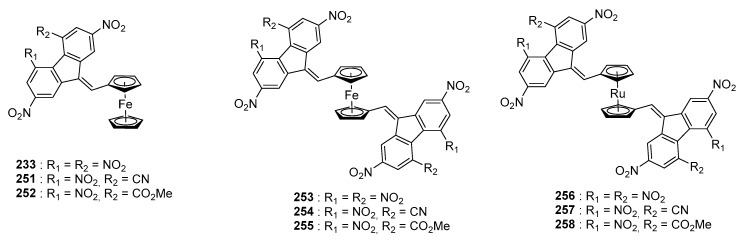
Push–pull chromophores based on metallocenes (iron and ruthenium complexes).

Finally, in 2001, the same authors investigated the design of A-D-A triads **233**, **251**–**258** where ferrocene or a ruthenium complex was used as the donor (See Figure 22) [101]. In these series, the higher electron donating ability of ferrocene compared to the ruthenium complex was demonstrated, a red-shift of the ICT band being detected for **253** (622 nm in 1,2-dichloroethane) compared to **256** (521 nm in 1,2-dichloroethane). Comparison of the UV-visible spectra of the triads **253**–**255** with the diads **233**, **251**, **252** revealed the position of the ICT bands to remain almost unchanged. Face to these considerations and considering the difficulty to synthesize the triads, it can be concluded that the synthesis of the diads is sufficient and that the connection of the donor to an additional electron acceptor can’t improve its electron-donating ability irrespective of the electron-withdrawing ability of the acceptor.

## 6. Conclusions

In this review, an overview of the different dyes designed with the fluorene scaffold substituted with three or four nitro groups is presented. As detailed in this review, organic dyes have focused the main interest of researchers, but metal-based chromophores have also been investigated. If poly(nitro)fluorene acceptors constitute a unique class of acceptor by their inherent absorption in the near infrared region, the presence of numerous nitro group drastically limits the solubility of the resulting push–pull dyes and numerous efforts have been devoted to overcome this drawback. At present, the scope of applicability of these structures is quite limited, since the compounds have mostly been investigated as sensitizers for hologram recording, and more scarcely for NLO or electrochromic applications. With regards to the recent interest of compounds absorbing in the near infrared region (photopolymerization, dyes for photovoltaics applications, etc.), clearly, the number of applications involving the use of poly(nitro)fluorene structures will greatly expand in the future.

## Figures and Tables

**Table 1:** UV-visible absorption characteristics of compounds **27**–**32** in *N*,*N*-dimethylformamide (DMF).

Compounds	27	28	29	30	31	32
ICT Bands (nm)	500, 587	492, 604	498, 613	509, 633	447, 599	446, 602

**Table 2:** UV-visible absorption characteristics of compounds in dioxane.

**Compounds**	**38**	**39**	**40**	**41**	**42**	**43**	**31**
ICT Bands (nm)	406, 491	412, 492	418, 515	420, 525	426, 556	426, 552	434, 571
**Compounds**	**44 ^1^**	**45 ^1^**	**46**	**47**	**48**	**49**	**32**
ICT Bands (nm)	403, 490	410, 514	416, 516	422, 526	425, 556	425, 554	433, 571
**Compounds**	**50**	**51**	**52**	**27**	**29**	**53**	**30**
ICT Bands (nm)	460, 527	470, 557	470, 565	480, 572	490, 592	486, 593	492, 606
**Compounds**	**28 ^2^**	**55**	**56**	**57**	**58**	**59**	
ICT Bands (nm)	492, 604	498	513	542	549	425, 569	

^1^ in acetone. ^2^ in DMF. ICT: intramolecular charge transfer.

**Table 3:** UV-visible absorption characteristics of compounds **71**, **77**, **83**, **90** and **95**–**97**.

Compounds	71	77	83	90	95	96	97
ICT Bands (nm) ^1^	544	592	611	588	636	707	753
ICT Bands (nm) ^2^	535	578	595	572	590	650	700

^1^ in 1,2-dichloroethane. ^2^ in acetone.

**Table 4:** UV-visible absorption characteristics of **84** and **106**.

Compounds	106 ^1^	84 ^2^
ICT Bands (nm)	545, 643	450, 610

^1^ in dichloromethane. ^2^ in acetone.

**Table 5:** Summary of the electrochemical data in dimethylacetamide (DMA) (vs. Fc^0^/Fc^+^ couple) and optical properties of the different dyes recorded in CH_2_Cl_2_.

Compounds	E_ox1_ (V)	E_red1_ (V)	E_red2_ (V)	E_red3_ (V)	E_red4_ (V)	λ_ICT_ (nm)
**84**	0.86, 1.22 irr ^1^	−0.91 ^1^	−1.10 ^1^	−1.91 ^1^	-	590
**84**	-	−0.80	−1.04	−2.04	-	
**83**	0.85 irr	−0.83	−1.07	−2.01	-	611
**82**	0.84 irr	−0.94	−1.15	−2.04	-	589
**81**	0.82, 1.35 irr ^1^	−1.11 ^1^	−1.21 ^1^	−1.89 ^1^	-	580
**81**	0.79 irr	−0.98	−1.22	−2.05	-	
**80**	0.76 irr	−1.12	−1.35	−2.02	-	554
**79**	0.73 irr	−1.18	−1.37	-	-	540
**78**	-	−1.28	−1.43	-	-	535
**77**	0.79 irr	−0.78	−1.02	−2.00	-	592
**76**	0.77 irr	−0.90	−1.11	−2.03	-	572
**75**	0.75 irr	−0.94	−1.18	−2.05	-	568
**74**	0.73 irr	−1.08	−1.29	−2.00	-	538
**73**	0.72 irr	−1.14	−1.32	−1.53 irr	-	529
**72**	-	-	-	-	-	498
**110**	-	−0.76	−1.01	−1.99	-	558
**111**	1.18 irr	−0.77	−1.02	−2.00	-	557
**71**	-	−0.76	−0.98	−1.79	−2.08	544
**70**	-	−0.87	−1.06	−1.81	−2.10	528
**69**	-	-	-	-	-	520
**68**	-	−1.05	−1.25	−1.83	−2.03	496
**67**	-	−1.11	−1.31	−1.88	-	488
**66**	-	-	-	-	-	472

^1^ in dichloromethane.

**Table 6:** UV-visible absorption characteristics of push–pull molecules **115**–**131**.

**Compounds**	**115**	**116**	**117**	**118**	**119**	**120**
ICT Bands (nm) ^1^	429	446	474	492	492	492
**Compounds**	**121**	**122**	**123**	**124**	**125**	**126**
ICT Bands (nm) ^1^	492	508	538	542	540	562
**Compounds**	**127**	**128**	**129**	**130**	**131**	
ICT Bands (nm) ^1^	502	520	537	574	596	

^1^ in DMF.

**Table 7:** UV-visible absorption characteristics of push–pull molecules **158**, **160**–**164**, and **169**.

**Compounds**		**168**		**158**	**169**
ICT bands (nm) ^1^		503		565	589
**Compounds**	**160**	**161**	**162**	**163**	**164**
ICT bands (nm) ^1^	489	513	530	567	588

^1^ in DMF.

**Table 8:** Summary of the electrochemical data in acetonitrile (vs. Fc^0^/Fc^+^ couple) and optical properties of the different dyes **210**–**224** recorded in dioxane.

Compounds	E_ox1_ (V) ^1^	E_red1_ (V) ^1^	E_red2_ (V) ^1^	E_red3_ (V) ^1^	E_red4_ (V) ^1^	λ_ICT_ (nm) ^2^
**210**	1.23	−0.89	-	-	-	441
**211**	1.33	−0.82	-	-	-	454
**212**	1.33	−0.79	-	-	-	460
**213**	1.34	−077	-	-	-	459
**214**	1.36	−0.70	−0.77	-	0.07	465
**215**	1.37	−0.67	−0.75	−1.32	0.08	472
**216**	1.47	−0.53	−0.62	−1.34	0.09	492
**217**	1.55	−0.40	−0.50	−1.31	0.10	505
**218**	0.71	−1.16	-	-	-	528
**219**	-	-	-	-	-	532
**220**	0.77	−1.04	−1.14	-	0.10	543
**221**	0.77	−1.03	1.13	-	0.10	542
**222**	0.87	−0.97	−1.06	-	0.09	539
**223**	0.89	−0.93	−1.03	−1.44	0.10	552
**224**	1.00	−0.84	−0.96	−1.50	0.12	564
**225**	1.11	−0.70	−0.86	−1.50	0.16	571

^1^ in acetonitrile. ^2^ in dioxane.

**Table 9:** Summary of the electrochemical data in CH_2_Cl_2_ (vs. Fc^0^/Fc^+^ couple) and optical properties of the different dyes **198**–**203** recorded in CH_2_Cl_2_.

Compounds	E_ox1_ (V)	E_ox2_ (V)	E_red1_ (V)	E_red2_ (V)	E_red3_ (V)	λ_ICT1_ (nm)	λ_ICT2_ (nm)
**227**	−0.13	0.37	−0.68	−1.04	−1.86	750	990
**228**	−0.09	0.35	−0.39	−0.90	−1.57	800	1230
**229**	−0.17	0.31	−0.69	−1.06	−1.84	775	975
**230**	−0.10	0.32	−0.38	−0.90	−1.56	800	1260
**231**	−0.11	0.40	−0.72	−1.00	−1.81	630 ^1^	900 ^1^
**232**	−0.10	0.41	−0.39	−0.93	−1.62	785–825	1200–1330

^1^ in acetone.

**Table 10:** Summary of the optical properties of the different dyes recorded in 1,2-dichloroethane.

**Compounds**	**233**	**234**	**236**	**238**	**239**	**241**
λ_ICT1_ (nm)	430	410	451	431	430	430
λ_ICT2_ (nm)	620	609	658	616	600	600
**Compounds**	**244**	**247**	**248**	**249**	**250**	
λ_ICT1_ (nm)	502	450	464, 514	450, 485	455, 490	
λ_ICT2_ (nm)	640	-	706	660	660	
